# Inhibition of Orbivirus Replication by Fluvastatin and Identification of the Key Elements of the Mevalonate Pathway Involved

**DOI:** 10.3390/v13081437

**Published:** 2021-07-23

**Authors:** Fauziah Mohd Jaafar, Baptiste Monsion, Mourad Belhouchet, Peter P. C. Mertens, Houssam Attoui

**Affiliations:** 1 UMR Virologie 1161, INRAE, Ecole Nationale Vétérinaire d’Alfort, ANSES, Université Paris-Est, 94700 Maisons-Alfort, France; fauziah.mohd-jaafar@vet-alfort.fr (F.M.J.); baptiste.monsion@vet-alfort.fr (B.M.); 2 Division of Structural Biology, Henry Wellcome Building for Genomic Medicine, Oxford OX3 7BN, UK; mourad.belhouchet@outlook.com; 3 Sutton Bonington Campus, School of Veterinary Medicine and Science, University of Nottingham, Leicestershire LE12 5RD, UK; peter.mertens@nottingham.ac.uk

**Keywords:** bluetongue virus, BTV, orbivirus, antiviral, fluvastatin, HMG-CoA reductase, IFNAR^(−/−)^ mice

## Abstract

Statin derivatives can inhibit the replication of a range of viruses, including hepatitis C virus (HCV, *Hepacivirus*), dengue virus (*Flavivirus*), African swine fever virus (*Asfarviridae*) and poliovirus (*Picornaviridae*). We assess the antiviral effect of fluvastatin in cells infected with orbiviruses (bluetongue virus (BTV) and Great Island virus (GIV)). The synthesis of orbivirus outer-capsid protein VP2 (detected by confocal immunofluorescence imaging) was used to assess levels of virus replication, showing a reduction in fluvastatin-treated cells. A reduction in virus titres of ~1.7 log (98%) in fluvastatin-treated cells was detected by a plaque assay. We have previously identified a fourth non-structural protein (NS4) of BTV and GIV, showing that it interacts with lipid droplets in infected cells. Fluvastatin, which inhibits 3-hydroxy 3-methyl glutaryl CoA reductase in the mevalonic acid pathway, disrupts these NS4 interactions. These findings highlight the role of the lipid pathways in orbivirus replication and suggest a greater role for the membrane-enveloped orbivirus particles than previously recognised. Chemical intermediates of the mevalonic acid pathway were used to assess their potential to rescue orbivirus replication. Pre-treatment of IFNAR^(−/−)^ mice with fluvastatin promoted their survival upon challenge with live BTV, although only limited protection was observed.

## 1. Introduction

The genus *Orbivirus* contains 22 virus species which are primarily vectored by *Culicoides* midges, ticks, phlebotomine flies, anopheline or culicine mosquitoes. Viruses belonging to the three economically most important orbivirus species: *Bluetongue virus* (BTV) (the type-species), *Epizootic hemorrhagic disease virus* (EHDV) and *African horse sickness virus* (AHSV) are all transmitted by *Culicoides* biting-midges, collectively infecting wild and domesticated ruminants and camelids or equids [1]. Several orbiviruses can also infect humans, including members of the *Changuinola virus*, *Corriparta virus*, *Lebombo virus*, *Orungo virus* and *Great Island virus* species [2]. Orbiviruses are emerging pathogens throughout the globe, as demonstrated by recent disease outbreaks in Europe, the detection of ‘novel’ BTV serotypes [3,4] and the emergence of novel orbiviruses such as *Peruvian horsesickness virus* in Australia and Peru [5].

Live attenuated BTV and AHSV vaccines were originally developed in South Africa but have more recently been superseded by inactivated vaccines against BTV types 1, 2, 4 and 8 in Europe, which are very effective, safer and compatible with DIVA PCR assays [6]. Passive transfer of AHSV-specific antibodies can also protect infected individuals against clinical signs of disease [7], and several compounds have been shown to inhibit dsRNA virus replication, including nucleoside analogues such as ribavirin triacetate, 3-deazaguanine and 3’-fluoro-3’-deoxyadenosine (3’F3’dAdo), which interfere with the replication of Colorado tick fever virus [8]. The nucleoside analogues 3-deazaguanine and 3-deazauridine or the aminothiophenecarboxylic acid derivative ‘compound 003′ (C003) can also interfere with BTV replication [9]. In a recent study, a compound known as aurintricarboxylic acid was shown to inhibit *Orbivirus* replication in both mammalian and insect cells [10]. However there are no commercially available and recommended antiviral treatments for orbiviral infections.

Until recently, only seven structural proteins (VP1-VP7) and three non-structural proteins (NS1-NS3 and NS3a) had been identified as encoded by the orbivirus genome [11]. A fourth non-structural protein designated NS4 was found, encoded by an alternate open reading frame in genome segment 9. This protein localises to the nucleoli and associates with lipid droplets in the infected-cell cytoplasm [12,13].

Four types of BTV particle have been described, including the core, infectious subviral particles (ISVP: generated by proteolytic cleavage of the outer capsid proteins), intact virus particles and transient membrane-enveloped intact viral particles (MEVP) [14,15]. During the late stages of infection, NS4 is associated with the cell-membranes of BTV-infected cells. The existence of MEVP and interactions of NS4 with lipid droplets suggest involvement of lipid pathways in orbivirus replication, particularly for BTV.

The 3-hydroxy-3-methylglutaryl-CoA (HMG-CoA) reductase enzyme is involved in the mevalonate pathway [16], converting HMG-CoA into mevalonic acid, and is competitively inhibited by the statin derivatives fluvastatin and lovastatin, which have structural similarities to HMG-CoA [16]. Downstream reactions that are affected by statins include the cascade of mevalonic acid, leading to the synthesis of farnesyl pyrophosphate (FPP). Farnesyl-PP is a ‘hub’ molecule that is used in the biosynthesis of ubiquinone, dolichols, Heme A and sterols [17]. Two additional pathways driven by farnesyl-PP, include the biosynthesis of cholesterol from squalene and the prenylation of proteins either by farnesylation or by geranylgeranylation [17]. Prenylation (transfer of farnesol or geranylgeraniol onto C-terminal cysteine moieties of a protein) is catalysed by farnesyl transferase (which can be blocked by farnesyl transferase inhibitors), or by geranylgeranyl transferase (which can be blocked by geranylgeranyl transferase inhibitors). In insects, the sterol branch of the mevalonate pathway is lost due to absence of the enzyme squalene synthase [18].

The statin derivative, ceestatin, inhibits HCV replication by blocking the synthesis of HMG-CoA synthase involved in the synthesis of HMG-CoA from acetoacetyl-CoA [19]. This mimics the effect of inhibitors of HMG-CoA synthase, such as hymeglusin [20]. The statin derivatives fluvastatin and lovastatin also interfere with replication of enveloped viruses such as HCV, dengue virus (DENV), West Nile virus, respiratory syncytial virus and African swine fever virus and non-enveloped viruses such as poliovirus [21,22,23,24]. However, the inhibitory effect of these statin derivatives on hepatitis C or dengue virus replication can be reversed by addition of chemical intermediates which restore the mevalonate pathway, including, mevalonate (such as mevalonolactone) or HMG-CoA. Supplementation of statin-treated cells with intermediate compounds that occur downstream of mevalonic acid, such as geranylgeraniol, also reverse the effect of statins on HCV and partially on DENV [25]. Although cholesterol or farnesol did not reverse the inhibitory effects of statins on the replication of HCV and HIV [26], farnesol did partially reverse the effect of statins on DENV replication. The inhibition of DENV replication by lovastatin also occurred when the squalene synthase (SQS) was inhibited by zaragozic acid A but not by farnesyl transferase inhibitor [27]. Lovastatin decreases the viral load of HIV but this effect can be reversed by addition of mevalonic acid and geranylgeraniol but not by cholesterol or farnesol [28]. 

Statin therapy is known to prevent geranylation of small G-proteins and to suppress inflammatory signals initiated from the plasma membrane [29,30]. This paper assesses the effect of fluvastatin on the replication of a *Culicoides*-borne orbivirus (bluetongue virus (BTV)), and a tick-borne orbivirus (Great Island virus (GIV)) in cell-cultures and during lethal virus-challenge of IFNAR^(−/−)^ mice.

## 2. Materials and Methods

### 2.1. Lipid Depletion of Foetal Bovine Serum (FBS)

Fumed silica (0.007µ, Sigma) was used to deplete lipids from foetal bovine serum (FBS) [31,32]. Serum was incubated with 20 mg of fumed silica per mL of serum and incubated overnight. The mixture was centrifuged at 2000× *g* for 15 min and serum was recovered, then treated with an equal volume of the Vertrel XF (Sigma), shaken and then centrifuged at 2000× *g* for 15 min. The supernatant was collected and used as a source of FBS in cell culture to assess the effect of lipid depletion on virus yields.

### 2.2. Chemical Compounds

Fluvastatin sodium (Sigma), the prenylation enzyme inhibitor (geranylgeranyl transferase inhibitor (GGTI-2133, Sigma), farnesyl pyrophosphate transferase inhibitor (FPTI III, Santa Cruz biotechnologies)) and the squalene synthase inhibitor zaragozic acid A (Sigma) were dissolved in DMSO and stored at −20 °C. Cholesterol (Sigma), mevalonic acid (Sigma) and geranylgeranyl pyrophosphate (GGPP, Sigma) were dissolved in absolute ethanol and stored at −20 °C. Farnesyl pyrophosphate (FPP, Sigma) was dissolved in methanol and stored at −20 °C.

Stock solutions were diluted in cell culture media to a final concentration of DMSO of 0.1% and ethanol or methanol of 0.2%.

### 2.3. Dosage of Fluvastatin in Mice

The oral LD50 of fluvastatin in mice is about 2 g/kg body weight, while the intraperitoneal LD50 is 0.14 g/kg body weight. The human therapeutic dose of fluvastatin is 0.6 to 1.2 mg/kg body weight [33]. The mouse dose is calculated by multiplying the human dose by 12.3. This type of conversion is known as interspecies allometric scaling and takes into account the differences in body surface area in m^2^ and animal weight [34]. Accordingly, the higher end human dose of 1.2 mg/kg dose results in a mouse dose of 14.8 mg/kg body weight (350 µg/mouse, assuming a mouse weighs 23–25 g). In humans, the maximum serum concentration of fluvastatin with a dose of 0.6 mg/kg body weight is about 530 ng/mL (±360), while with a dose of 1.2 mg/kg, it is 1420 ng/mL (±880) [33].

With a molecular weight of 433, a fluvastatin serum concentration of 530 ng/mL corresponds to ~1.25 µM and a concentration of 1420 ng/mL corresponds to ~3 µM.

In cell cultures, the inhibitory dose of fluvastatin for flaviviruses such as Zika virus is approximately 3 µM [35].

### 2.4. Cell Lines and Viruses

BSR cells [36] were grown at 37 °C in Dulbecco’s minimum essential medium (DMEM) supplemented with 10% FBS, penicillin G (100 IU/mL) and streptomycin (100 µg/mL).

BSR cells were used to propagate bluetongue virus (BTV-8 NET2006/08: orbivirus collection Pirbright, UK), Great Island virus (GIV: kindly provided by Robert Tesh, UTMB, Galveston, TX, USA) and yellow fever virus 17D strain (YFV17D: kindly provided by Robert Tesh, UTMB, Galveston, TX, USA). Plates with 48 wells were seeded with 2 × 10^5^ BSR cells/well.

*Culicoides sonorensis*-derived KC cells [37] were grown at 28 °C in Schneider’s medium supplemented with 10% FBS, penicillin G (100 IU/mL), streptomycin (100 µg/mL) and 10% tryptose phosphate broth. Plates with 48 wells were seeded with 7 × 10^5^ BSR cells/well.

Infection of BSR cells with YFV17D was performed using 0.1 PFU of virus/cell. BSR or KC cells were infected with orbiviruses at 0.05 PFU/cell. Briefly, cell monolayers were washed once with serum-free medium, incubated with 250 µL of virus suspension for 3 h. Assay cells were pre-treated with fluvastatin, zaragozic acid A, FPTI III and/or GGTI-2133 for 24 h prior to infection. The supernatant was discarded and replaced with fresh culture medium, containing the same chemical used for pre-treatment. Chemicals including mevalonic acid, geranylgeranyl pyrophosphate, farnesyl pyrophosphate or cholesterol, were added to cell culture upon replenishing with fresh culture medium.

### 2.5. Plaque Assay

BSR cells were ‘plated’ a day before the plaque assay [38,39]. A 10-fold serial dilution of virus suspension was prepared in serum-free DMEM. Confluent BSR cell-monolayers were inoculated with 250 µL of diluted virus suspensions and incubated at 37 °C for 2 h. The supernatant was then discarded, and the cells washed once with serum-free DMEM. The medium was then discarded and replaced with molten 1% low melting point agarose (Sigma) in DMEM. Plates were subsequently incubated at 37 °C for 5 days, fixed by addition 10% formaldehyde in phosphate-buffered saline per well. After removal of agarose plugs, monolayers were stained with 0.1% naphthalene-black solution, then washed with deionised water and the plaques counted.

Log reduction of virus titre, as a result of treating with an inhibitor, is calculated from the formula log_red_ = log_10_(A) − log_10_(B), where A and B are the virus titres of non-treated cell and treated cells, respectively. To express log reduction as a percentage, the following formula is used *p* = (1 − 10^−L^) × 100, where *p* is the percentage reduction and L is the log_10_ reduction.

### 2.6. Cytotoxicity of Fluvastatin Treatment Assessed by an MTT Cell Proliferation Assay

In order check that changes in viral yields are not simply due to a cytotoxic effect but are caused a pharmacological effect on virus replication, we used 3-(4,5-dimethylthiazol-2-yl)-2,5-diphenyl tetrazolium bromide (MTT, Roche diagnostic) to assess the cytotoxicity of treatment with fluvastatin. For this purpose, 2.5 × 10^4^ BSR cells or 5 × 10^4^ KC cells were seeded into 96-well culture plates. Cell culture medium was replaced with a fresh medium containing 2% FBS. Fluvastatin at 1.25, 2.5, 5, 10 and 20 µM were added to different wells in duplicates. The mean values of four independent experiments was reported. Following incubation with fluvastatin, cell culture medium was removed and cytotoxicity was assessed using an MTT cell proliferation kit I (Roche diagnostics), as described by the manufacturer. Cell viability was expressed as a percentage compared to an untreated BSR or KC control: ([absorbance treated cells]/[absorbance control cells]) × 100.

### 2.7. Antibodies to Great Island Virus (GIV) NS4, Bluetongue Virus 8 Fourth Non-Structural Protein (BTV-8 NS4) and BTV-8 VP2

Rabbit antibodies against NS4 of BTV-8 and GIV and mouse antibodies against VP2 of BTV-8 were previously described [12,39]. These antibodies were used to assess levels of protein expression at 48 h post-infection by BTV or GIV by confocal immunofluorescence imaging as previously described [12].

### 2.8. RNA Extraction and Real-Time Polymerase Chain Reaction (PCR)

Levels of virus replication in chemically-treated and untreated infected cells was assessed by plaque assay on BSR cells and/or by real-time RT-PCR of RNA extracted from the cells. Biological triplicates of virus infected BSR cells from four independent experiments were harvested 40 h post-infection. Biological triplicates of BTV-8 infected KC cells from 3 independent experiments were harvested at day 5 post-infection. Cells were scraped and centrifuged at 2000× *g* for 10 min and pellets were extracted with TRIzol (Thermofisher) as previously described [40].

In mice, viraemia was assessed by real-time RT-PCR of RNA extracted from blood. Real-time RT-PCR for orbiviruses was performed using primers and probes targeting genome segment 10 of BTV (BTV_S10_F: TGGAYAAAGCRATGTCAAA, BTV_S10_P: FAM-ARGCTGCATTCGCATCGTACGC-BHQ1, BTV_S10_R: ACRTCATCACGAAACGCTTC) [41] or genome segment 10 of GIV (GIV_S10_F: CGACGCGTATGCTCAAG, GIV_S10_P: FAM-TGCAGTCTTACGGAGATGCG-BHQ1, GIV_S10_R: CAGCTAGACACAGCAAC). A 3.3 Ct difference corresponds to a 10-fold (or 1 log) change in viral RNA copies.

### 2.9. Identification of Culicoides 3-Hydroxy-3-Methylglutaryl-CoA (HMG-CoA) Reductase

Insect HMG-CoA reductase is inhibited by fluvastatin and inhibition can be reversed in insect cells by addition of mevalonic acid [42,43,44].

*Culicoides* HMG-CoA reductase was identified using the online tblast-n software implemented in VectorBase (www.vectorbase.org, accessed on 2 June 2021). HMG-CoA reductase from different organisms was downloaded and relatedness to other arthropod HMG-CoA reductases was assessed using MEGA X [45].

### 2.10. Pharmacological Inhibition of Virus Replication

#### 2.10.1. Yellow Fever Virus 17D Strain

We used yellow fever virus 17D (genus *Flavivirus*, family Flaviviridae) as a paradigm and a control to assess the effect of fluvastatin on virus replication. Earlier studies have shown that lipid depletion of serum used in culture medium led to inhibition of dengue virus [46]. Replication of West Nile virus depends on the cholesterol biosynthesis pathway and consequently inhibiting cholesterol and alteration of cellular geranylgeranylated proteins limit virus replication [47]. BSR cells were grown in 24 well plates. At 48 h prior to infection, culture medium was replaced with fresh medium containing 2% normal FBS or lipid-depleted FBS. Infections with YFV17D were carried out in the absence or presence of fluvastatin (3 µM), which was added to the cells 24 h before infection.

Zaragozic acid A (ZGA) is an inhibitor of squalene synthase [26,48]. ZGA was previously shown to inhibit dengue virus (*Flavivirus*) replication. We used ZGA (10 µM) which was added 24 h before infection to BSR cell monolayers and maintained during infection with YFV17D.

The effects of FPTI III, a farnesyl pyrophosphate transferase inhibitor (FPTI) and GGTI-2133 a geranylgeranyl transferase inhibitor (GGTI) on YFV replication were also assessed. BSR cells were treated with 30 µM of either FPTI III or GGTI-2133. Twenty-four hours later the cells were infected with 0.05 PFU/cell of YFV17D, in the presence of the FPT or GGT inhibitors. The inoculum was removed and replaced with fresh culture medium supplemented with either FPTI III or GGTI-2133. At 40 h post-infection (p.i.) cells were scraped off and mixed supernatant and cells Dounce-homogenised before being titrated in plaque assays.

Mevalonate pathway intermediates, including mevalonic acid (200 µM), GGPP (25 µM), FPP (25 µM) or cholesterol (5 µg/mL) were added in cell culture medium, to cells infected with YFV17D and pre-treated with fluvastatin, to assess their capacity to rescue virus replication.

#### 2.10.2. Orbiviruses (BTV-8 and GIV) in Mammalian and/or Insect Cells

BSR cells (grown in a 12-well plate containing a 16 mm cover slide) were pre-treated with fluvastatin at 1.25 µM (final concentration in cell culture medium) for 24 h before being infected with BTV-8 or GIV. At 48 h post-infection, cells were fixed with paraformaldehyde for immunofluorescence analysis. NS4 or VP2 expression was assessed by confocal immunofluorescence using rabbit anti-NS4 antibodies (BTV-8 and GIV) or mouse anti-VP2 antibodies (BTV-8). BTV-8 replication in KC cells and BTV-8 or GIV replication in BSR cells, infected at a multiplicity of infection (MOI) of 0.05 PFU/cell, was also assessed by real-time RT-PCR and/or plaque assay.

Treatment with ZGA (10 µM), FPTI III (30 µM), GGTI-2133 (30 µM) was performed as described above. Mevalonic acid (200 µM), GGPP (25 µM), FPP (25 µM), or cholesterol (5 µg/mL) were added to orbivirus infected cells that had been pre-treated with fluvastatin, to assess the effect of adding pathway intermediates. At 40 h post-infection, cells were scraped from the well. Mixed cells and supernatant were extracted using TRIzol, or Dounce-homogenised. The level of virus replication was assessed using plaque assay and TaqMan real-time RT-PCR.

### 2.11. Treatment of IFNAR^(−/−)^ Mice with Fluvastatin and Infection with BTV

IFNAR^(−/−)^ mice (genetic background: A129SvEvBrd) were a gift from Professor Michel Aguet (ISREC, Ecole Polytechnique Fédérale de Lausanne, Switzerland). IFNAR^(−/−)^ mice can be lethally infected with orbiviruses, in particular BTV [39,49]. Six- to eight-week-old IFNAR^(−/−)^ mice were used throughout the experiments. Six groups of five IFNAR^(−/−)^ mice were constituted as shown in Table 1. Blood (30 µL/mouse) was sampled from the retro-orbital sinus of animals on days 0, 4, 6, 8, 12 and 15. Total RNA was extracted from 10 µL of blood using TRIzol as described.

### 2.12. Statistical Analyses

Statistical analyses were performed using jamovi (Version 1.8; https://www.jamovi.org accessed on 18 May 2021).

## 3. Results

### 3.1. Identification of Culicoides HMG-CoA Reductase

Using the HMG-CoA reductase EC number 1.1.1.34, the protein sequences corresponding to *Homo sapiens* and *Drosophila melanogaster* enzymes were retrieved (https://www.ncbi.nlm.nih.gov/gene/3156; https://www.ncbi.nlm.nih.gov/gene/42803, accessed on 2 June 2021).

Among the data associated with the sequences are the links to conserved domain structures (https://www.ncbi.nlm.nih.gov/Structure/cdd/cddsrv.cgi?uid=273339; https://www.ncbi.nlm.nih.gov/Structure/cdd/cddsrv.cgi?uid=153081, accessed on 2 June 2021). The consensus sequence of the HMG-CoA reductase was retrieved and used for the tblast-n search implemented in VectorBase.

**Figure 1:**
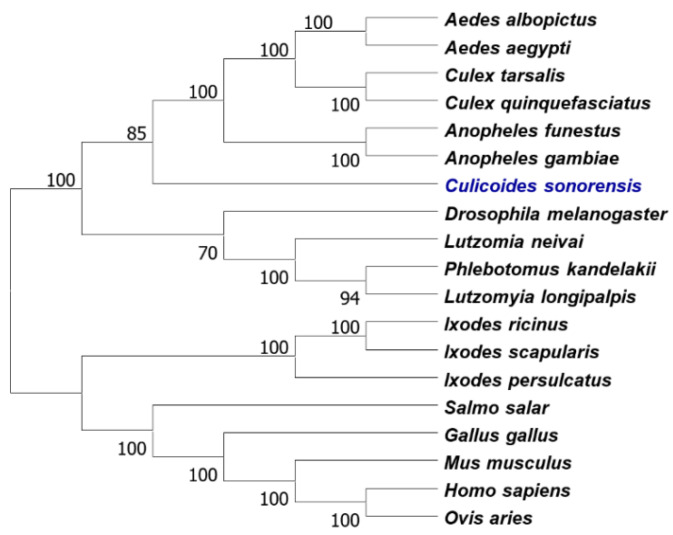
A neighbour-joining phylogenetic tree constructed using MEGA X, showing the clustering of *Culicoides sonorensis* 3-hydroxy-3-methylglutaryl-CoA (HMG-CoA) reductase amongst insect HMG-CoA reductase enzymes. Accession numbers of sequences used to construct the tree are provided in Supplemental Table S1.

Transcripts CSON008391, CSON008765 and GAWM01005591 were identified as encoding the HMG-CoA reductase of *Culicoides sonorensis*. Assembly of these three transcripts made it possible to generate a full-length aa sequence of the enzyme, which can also be found in scaffold LN483907 retrieved from Genbank database (Figure S1). A tree constructed using mosquito, sandfly, tick, mammalian, fish and avian HMG-CoA reductase is shown in Figure 1, indicating that the *Culicoides* enzyme clusters with other insect HMG-CoA reductases. The sequence of the *Culicoides sonorensis* HMG-CoA reductase is 50% identical (68% similar) to the human enzyme.

A structural model of the *Culicoides* enzyme was generated using Phyre2 protein fold recognition server (http://www.sbg.bio.ic.ac.uk/phyre2, accessed on 18 May 2021). We found that 422 residues (amino acids 434–856: 45% of the sequence) were modelled on the structure of the human HMG-CoA reductase with very high confidence (100%) and strong identity (58%). Residues 36–306 (29% of the sequence) were modelled with high confidence (100%) onto the intracellular cholesterol transporter 1 protein.

### 3.2. Cell Viability Assay in Presence of Fluvastatin

**Figure 2:**
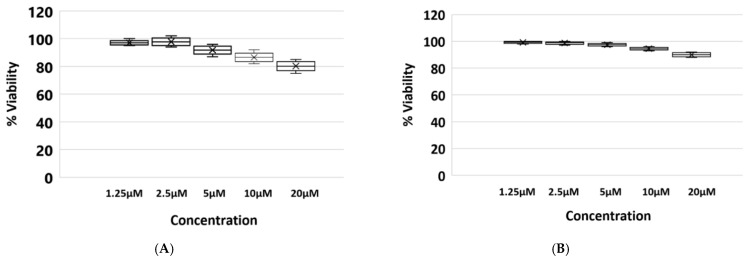
Effect of fluvastatin on cell viability. (**A**) Effect of increasing concentrations of fluvastatin on BSR cell viability. Cells were harvested at 72 h post-treatment and assessed by an MTT viability assay. (**B**) Effect of increasing concentrations of fluvastatin on KC cell viability. Cells were harvested on day 5 post-treatment.

BSR viability was assessed at 72 h post-treatment at a range of fluvastatin concentrations. No cytotoxic effects were detected at 1.25 µM or 2.5 µM fluvastatin. At 5 µM a minimal cytotoxicity was observed where cell viability was reduced by about 8%. The strongest cytotoxic effect was observed with concentrations of 10 and 20 µM, with cell viability reduced by 14 and 20% respectively (Figure 2A).

KC cells viability was also assessed on day 5 post-treatment with the same concentrations of fluvastatin used for BSR cells. Concentrations of 1.25 µM, 2.5 µM or 5 µM fluvastatin did not induce cytotoxic effects or affect cell viability. At 10 µM and 20 µM cell viability was reduced by approximately 5% and 10%, respectively, (Figure 2B).

### 3.3. Fluvastatin and Replication of Yellow Fever Virus YFV17D in BSR Cells

**Figure 3:**
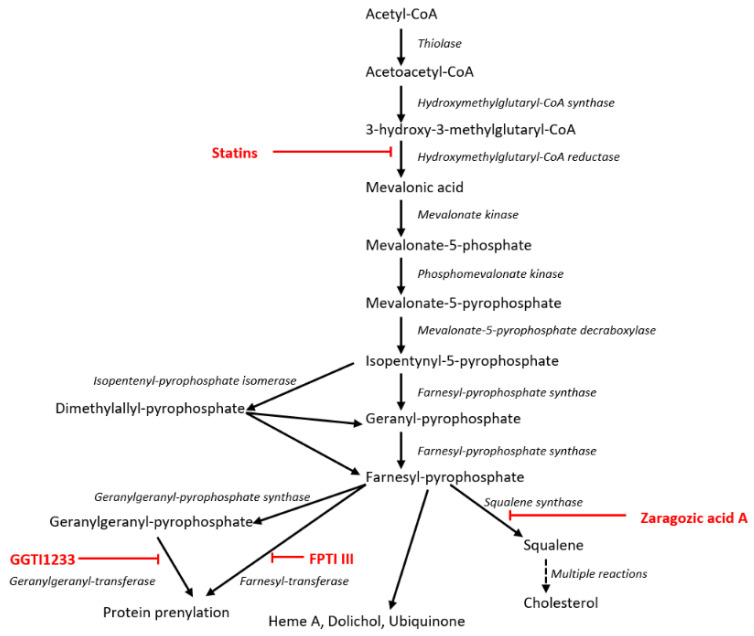
The mevalonate pathway of mammals. Key steps inhibited by statins (inhibition of HMG-CoA reductase), zaragozic acid A (inhibition of squalene synthase), geranyl-geranyl pyrophosphate or farnesyl pyrophosphate inhibitors are indicated.

In BSR cells, the mean titre of YFV17D at 40 h post/infection was ~4 × 10^7^ PFU/mL, as determined by a plaque assay [39]. Maintenance of cells in lipid-depleted medium, reduced YFV titres (by ~1 log_10_: 90% reduction), indicating that exogenous cholesterol is important for virus replication. The mevalonate pathway is summarised in Figure 3.

**Figure 4:**
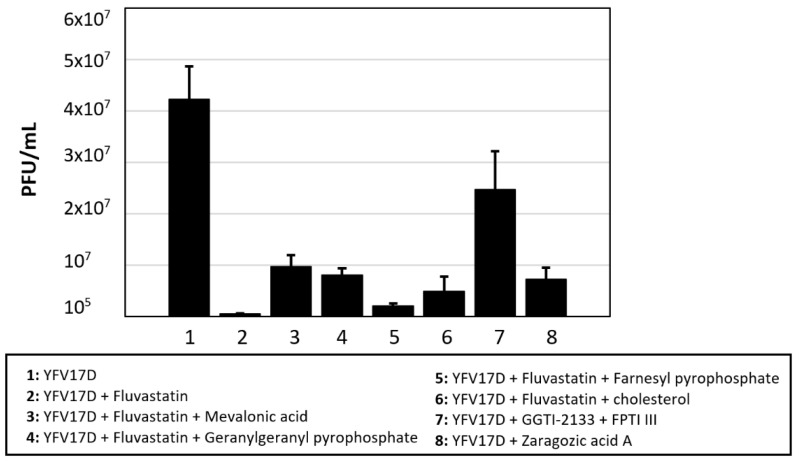
YFV17D-infected BSR cells treated with fluvastatin, inhibitors of geranyl-geranylation (geranyl-geranyl transferase inhibitor GGTI-2133 or farnesyl pyrophosphate transferase inhibitor FTPIII), inhibitors of squalene synthase (zaragozic acid), or treated with fluvastatin then supplemented with components of the mevalonate pathway (mevalonic acid, geranyl-geranyl pyrophosphate, farnesyl pyrophosphate or cholesterol) in attempts to restore virus replication in fluvastatin-treated cells. These results are representative of four distinct experiments. Error bars with standard deviations are shown.

Treatment of BSR cells with fluvastatin or ZGA also showed significant reductions in YFV17D titres (by ~1.8 or 0.8 log_10_, respectively (98% and 84% reduction of virus titres, respectively.) (Figure 4) [50].

Addition of cholesterol to the culture media of cells treated with fluvastatin led to a recovery of YFV titres by ~1 log_10_ (90% recovery, compared to fluvastatin-treated cells: Figure 4 and Table S2). Supplementation with mevalonic acid or GGPP also rescued YFV17D replication (by ~1.2 log or 99.7% recovery as compared to fluvastatin-treated cells which are not supplemented with mevalonic acid) while FPP had a more limited effect on YFV recovery (by ~0.6 log or 75% recovery). Treating cells with FPTI or GGTI did not cause any significant reduction in the YFV17D titres (Figure 4).

### 3.4. Fluvastatin and Replication of Orbiviruses in Mammalian Cells

**Figure 5:**
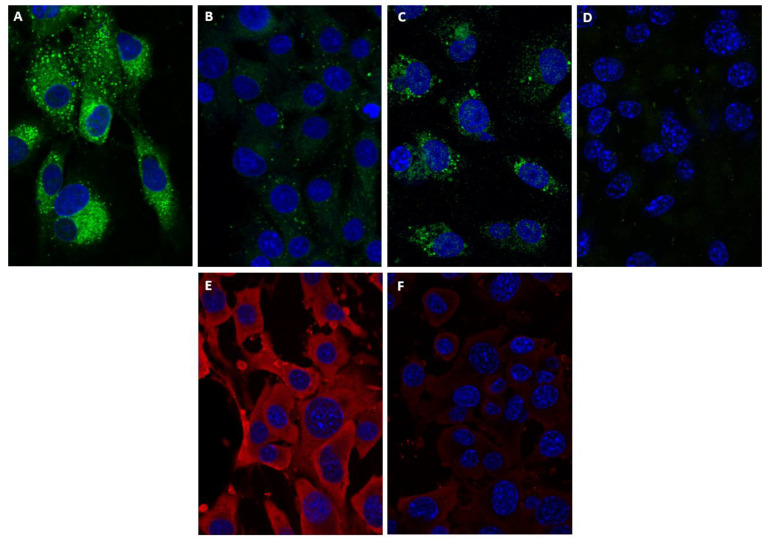
Confocal fluorescence microscopy of Great Island virus (GIV) or BTV infected BSR cells treated with fluvastatin. GIV infected cells were labelled with anti-GIV-NS4 (non-structural protein) and Alexa Fluor 488 (green fluorescence) conjugated anti-rabbit IgG. BTV infected cells were labelled with anti-BTV-NS4 and Alexa Fluor 488 (green fluorescence) conjugated anti-rabbit IgG or anti-BTV-8 VP2 and Alexa Fluor 568 (red fluorescence) conjugated anti-mouse IgG. Nuclei were stained with DAPI (blue). (**A**) Non-treated GIV infected cell control showing the NS4 spherical bodies. (**B**) GIV infected cells treated with fluvastatin, showing an almost complete disappearance of the NS4 spherical bodies and low levels of NS4 expression in the cells. (**C**) Non-treated BTV-8 infected-cell control showing NS4 in the cytoplasm and nucleus. (**D**) BTV infected cells treated with fluvastatin, showing an almost complete disappearance of the NS4 signal. (**E**) Non-treated BTV-8 infected-cell control showing strong expression of VP2 across the cells. (**F**) BTV-8 infected cells treated with fluvastatin, showing a significant drop of the VP2 signal.

Maintenance of BTV-8 infected BSR cells in lipid-depleted medium, showed a reduction in virus titres (~0.6 log: 75% reduction). In the absence of fluvastatin, NS4 was observed in both BTV-8 and GIV infected cells, associated with spherical bodies that were previously identified as lipid-droplets/NS4 complexes [12] (Figure 5A–D). However, fluvastatin treatment significantly reduced the amount of NS4 detected for both viruses, as well the level of VP2 of BTV-8 (Figure 5E,F), when compared to non-treated cells.

Significantly lower levels of BTV-8 RNA synthesis (reduced by 6.5 Ct (~2 log10) compared to untreated cells) were also detected by RT-PCR in infected cells treated with fluvastatin. Plaque assays also showed a 1.7 log10 reduction (98% reduction: *p* < 0.0001) in virus titre confirming that BTV replication was reduced after fluvastatin treatment.

**Figure 6:**
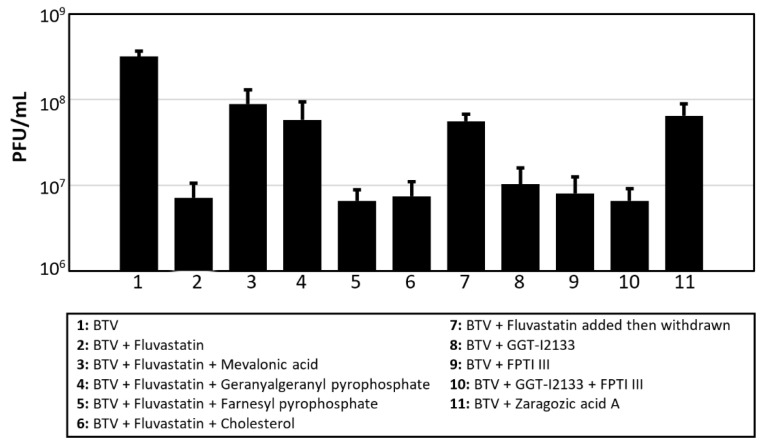
Effects of fluvastatin, mevalonate pathway-components and inhibitors on BTV replication in BSR cells. BTV-8 titres (PFU/mL) generated in BTV-8-infected BSR cells, treated with: fluvastatin; inhibitors of geranyl-geranylation (geranyl-geranyl transferase inhibitor GGTI-2133, or farnesyl pyrophosphate transferase inhibitor FTPIII); or inhibitors of squalene synthase (zaragozic acid A). Some cells were treated with fluvastatin then supplemented with components of the mevalonate pathway (mevalonic acid, geranylgeranyl pyrophosphate, farnesyl pyrophosphate or cholesterol), to inhibit, or in attempts to restore, virus replication in fluvastatin-treated cells. These results are representative of 4 independent experiments. Error bars with standard deviations are shown.

Addition of mevalonic acid (200 µM) or GGPP (25 µM) to the fluvastatin-treated, BTV infected cells reversed the inhibitory effect of fluvastatin, helping the BTV titre recover by 1.1 and 0.9 log10, respectively, (92% and 87% recovery, respectively: *p* < 0.0001 and 0.0053, respectively) (Figure 6 and Table S3). However, like HCV or HIV [26], addition of FPP (25 µM) did not help recovery of BTV replication. Treating BSR cells with FPTI III and/or GGTI-2133 prior to infection with BTV-8 reduced virus yields in a manner similar to fluvastatin treatment (1.6–1.7 log10 reduction: 97–98% reduction: *p* < 0.0001) (Figure 6 and Table S3). Treatment with ZGA also reduced BTV titre by ~0.7 log10 (80%: *p* < 0.0001).

Real-time RT-PCR also indicated significantly lower levels of GIV viral-RNA synthesis (by approximately 5.1 Ct (~1.5 log_10_)) in infected cells treated with fluvastatin.

### 3.5. Fluvastatin and Replication of BTV-8 in Culicoides KC Cells

**Figure 7:**
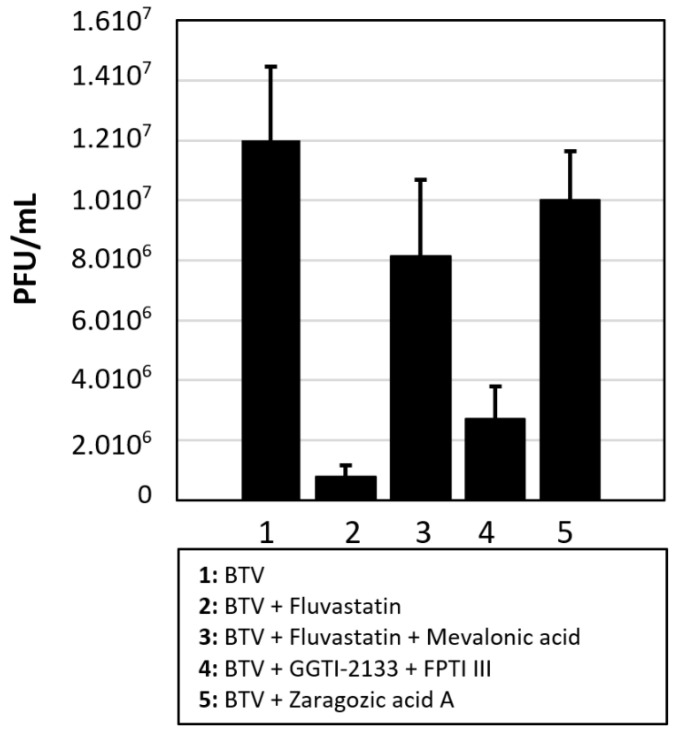
Effects of fluvastatin, mevalonate pathway inhibitors and components on BTV replication in KC cells. BTV-8 virus titres (PFU/mL) generated by infected KC cells treated with fluvastatin, inhibitors of geranylgeranylation (geranyl-geranyl transferase inhibitor GGTI-2133 and farnesyl pyrophosphate transferase inhibitor FTPIII), or inhibitors of squalene synthase (zaragozic acid A). Some cells were treated with fluvastatin then supplemented with mevalonic acid in attempts restore virus replication. These results are representative of three independent experiments. Error bars with standard deviations are shown.

Real-time RT-PCR results indicate significantly lower levels of viral RNA synthesis in BTV-8 infected KC cells treated with fluvastatin, with an approximately 4.6 Ct increase in RT-PCR assays (reduction of RNA levels of ~1.4 log_10_) compared to untreated cells (Figure 7, Table S4). This was confirmed by plaque assays showing a 1.2 log reduction of virus titre (93% reduction: *p* < 0.001), confirming BTV replication was reduced after fluvastatin treatment.

Rescue of virus replication in fluvastatin-treated KC cells, by addition of mevalonic acid (200 µM), indicates that fluvastatin also inhibits the *Culicoides* HMG-CoA reductase. Mevalonic acid helped BTV titre recovery (by 1 log_10_: 90% recovery, *p* = 0.002) (Figure 7 and Table S4). Treating KC cells with a combination of FPTI III and GGTI-2133 prior to infection with BTV-8 also reduced virus yields (0.7 log reduction: 80% reduction, <0.001) (Figure 7 and Table S4), suggesting that prenylation is also essential in insect cells. However, treatment of KC cells with ZGA did not cause any significant changes in virus titres (Figure 7, Table S4), suggesting that as in other insect cells, the sterol branch of the mevalonate pathway is non-functional [18].

### 3.6. Fluvastatin and Replication of Orbiviruses in IFNAR^(−/−)^ Mice

**Figure 8:**
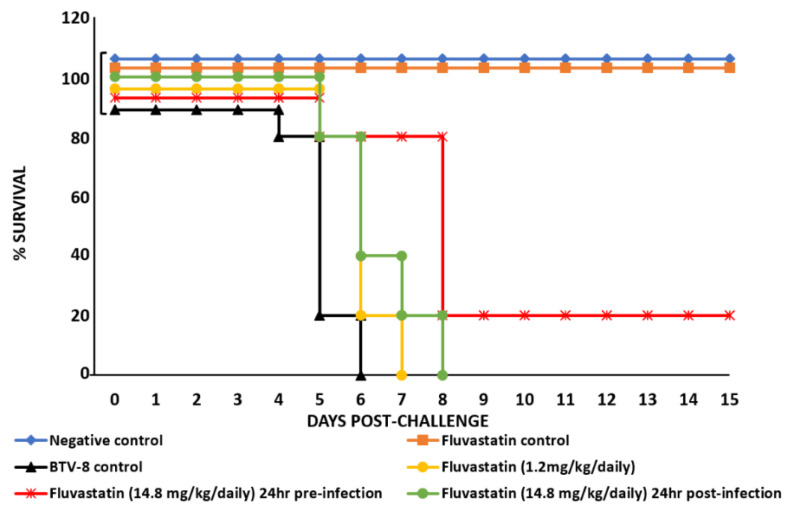
Survival curves of IFNAR^(−/−)^ mice.

IFNAR^(−/−)^ mice infected with BTV-8 were treated with fluvastatin to explore its effect on survival times post-challenge (p.c.), providing a relative measure of protection (Figure 8). RNA extracts of blood samples collected on days 0, 4, 6, 8, 12 and 15 p.c. from individual mice, were tested using a genome-segment-10 based real-time RT-PCR assay (Table S4). Uninfected negative-control and fluvastatin-control groups (A and C) remained negative for BTV throughout the experiment. Mice in the virus-infected control group (B) developed Ct values of ~25 to 28 on day 4 post challenge and only 1 animal survived until day 6 (when it was euthanised) with a Ct value of ~32. Animals infected with BTV-8 and treated with 1.2 mg/kg/day of fluvastatin (group D) did not show significant differences from the infected control group (group B) with Ct values of ~24 to 28, and all animals had died by day 7 p.c. However, mice in group (E), treated with 14.8 mg/kg/day fluvastatin (mouse-adapted dose) 24 h prior to virus challenge behaved differently, with higher. Ct values on day 4 p.c. (~30 to 34), indicating lower levels of viraemia. In this group, one animal died with the remaining 4 mice having Ct values of ~30 to 37 on day 6 p.c. Three of the surviving animals died, with the remaining mouse having a Ct value of 39.7 on day 8. This mouse recovered and survived until the end of the experiment with a Ct value of 40.8 on day 12 and was negative on day 15.

In a second separate experiment conducted using the same parameters, two of the mice in group E treated with fluvastatin 24 h prior to virus challenge, survived past day 8 until the end of the experiment on day 15, although one of these mice did show signs of reduced mobility. The mice in group F, treated with 14.8 mg/kg/day fluvastatin (mouse-adapted dose) but starting 24 h p.c., had Ct vales close to those of the untreated virus control group (~23 to 29). In this group, three mice died on day 6 and the remaining two mice had Ct values of 33.6 and 35.1. The mouse with a CT value of 33.6 on day 6, had a lower Ct value (28.4), showed severe clinical signs and was euthanised on day 8. The last mouse (with the higher Ct value) also died on day 8.

Six groups of 5 IFNAR^(−/−)^ mice were used as detailed in Table S5. The 350 µg/mouse oral dose of fluvastatin did not result in any apparent toxicity. Fluvastatin promoted survival in animals treated daily with an oral mouse adapted dose, when administered 24 h prior to challenge with live BTV-8.

## 4. Discussion

Statin derivatives show promise for the treatment of viral infections, possibly in combination with other therapeutic molecules with known antiviral effects (e.g., interferon alpha). Lovastatin and fluvastatin have been shown to interfere with the replication of HCV, dengue virus and African swine fever virus, with fluvastatin showing the strongest inhibitory effects on virus replication [19,21,23,25,26,27]. The effect of lovastatin on rotavirus replication has also been assessed, causing a 2-log_10_ reduction in virus titres [51].

We have previously shown that orbiviruses interact with the lipid pathways in mammalian cells and that orbivirus NS4 associates with lipid droplets in BTV- and GIV-infected cells [12]. In the current study, fluvastatin treatment of orbivirus-infected BSR cells significantly reduced the NS4 immunofluorescence-signal and almost abolished NS4 interactions with lipid droplets. However, the BTV-VP2 immunofluorescence-signal was also reduced, indicating a wider effect on virus replication. Quantification of viral RNAs in fluvastatin-treated, BTV-infected BSR cells, showed an increase of ~6.5 Ct in real-time RT-PCR assays, and BTV titres were reduced by 1.7 log_10_. Similar reductions were also observed in GIV RNA synthesis in fluvastatin-treated BSR cells (with an increase of 5.1 Ct).

We identified a full-length HGM-CoA reductase in *Culicoides sonorensis* (50% identical, 68% similar to the human HMG-CoA reductase), indicating a functional mevalonate pathway in KC cells. Fluvastatin treatment of BTV infected KC cells also reduced virus titres by 1.2 to 1.4 log_10_.

Our results with orbiviruses contrast with the findings of a rotavirus/lovastatin study [51]. Although rotavirus titres were also reduced by treatment with lovastatin, viral mRNA levels remained unchanged although there was an accumulation of viral proteins in infected cells. Observation by electron microscopy also indicated a reduction in progeny virus particle numbers, which is thought likely to be due to interference with virus assembly as a result of cholesterol depletion [51].

Infection of IFNAR^(−/−)^ mice with BTV can cause severe pathological changes, with viraemia detected by days 3–4 post-infection, leading to death by days 5–7 [39,52]. In the current study, treatment of IFNAR^(−/−)^ mice with different doses of fluvastatin, and the timing of administration, both influenced mice survival. A dose of 1.2 mg/kg/daily (comparable to a higher end human dose) did not protect the mice and did not reduce levels of virus replication. However, at a dose of 14.8 mg/kg/daily (obtained by multiplying the higher end human dose with 12.3 as explained in the materials and methods) promoted mouse survival, provided the treatment was initiated prior to BTV challenge. This resulted in a reduction in viraemia, as detected by higher Ct values, along with survival of one out of 5 mice.

These results are similar to those previously published in a study of dengue virus in IFNAR^(−/−)^ mice. Administration of lovastatin prior to DENV challenge reduced levels of viraemia and extended mice survival [53].

We used yellow fever virus (YFV17D) as a paradigm to assess the antiviral effects of fluvastatin. The effect on virus replication, particularly in cell cultures, was assessed by compensating for intermediates of the mevalonic acid pathway blocked by fluvastatin. Molecules including mevalonate and geranylgeranyl pyrophosphate restored the pathway rescuing both yellow fever virus and orbivirus replication. However, farnesyl pyrophosphate had only limited ‘rescue’ effect on YFV replication and no apparent effect on BTV replication.

We also endeavoured to differentiate between the cholesterol branch and the prenylation branch of the mevalonic acid pathway to identify whether either or both influence orbivirus replication. Supplementation of virus-infected and fluvastatin-treated cultures with cholesterol, rescued the replication of YFV but not that of BTV. Zaragozic acid A (ZGA) inhibits SQS in the cholesterol branch of the pathway, and reduces DENV replication in mammalian cells [25]. We observed a similar effect of ZGA on YFV17D replication in BSR cells, with titres reduced by ~0.8 log_10_ (84% reduction) in treated cells. Despite the lack of a cholesterol induced effect, ZGA also reduced BTV titre by ~0.7 log_10_ (80%), suggesting that the cholesterol-branch of the pathway has some influence on orbivirus replication although this may be relatively limited, as compared to fluvastatin treatment.

Treating BSR cells with inhibitors of geranylgeranyl transferase and farnesyl transferase (in the prenylation branch of the pathway) prior to virus infection had a minor effect on YFV replication but inhibited the replication of the orbiviruses at a similar level to fluvastatin, suggesting that inhibition of protein prenylation influences orbivirus replication. BTV-8 replication was inhibited in KC cells, by treatment with fluvastatin or a combination of GGTI-2133 and FPI III, suggesting that the mevalonic acid pathway is conserved in KC cells. However, treating KC cells with ZGA did not have any significant effect on BTV-8 replication, suggesting like in other insect cells the cholesterol branch of the pathway is absent or inactive in KC cells.

Replication of poliovirus (another non-enveloped virus) is inhibited by simvastatin, with the inhibitory effect attributed to reduced viral transcription, confirming that these effects are not limited to lipid-enveloped viruses [22].

In summary, the results presented here show that fluvastatin treatment of infected cell-cultures reduced progeny orbivirus titres by up to 1.7 log_10_ (98%). Inhibition of prenylation affects activities of prenylated proteins including Ras and Rho GTPases. Members of family *Reoviridae* such as the mammalian orthoreoviruses use the activated Ras pathway [54,55,56]. Transformed cells with constitutively activated Ras are highly permissive to orthoreovirus infection/replication. Ras activation results in inhibition of the dsRNA-dependent protein kinase (PKR), which is believed to enhance viral replication [54,55]. It is therefore likely that the inhibition of Ras prenylation in BSR cells leads to a reduced capacity of orbiviruses to efficiently infect/replicate in BSR cells. In addition, although survival of mice challenged with live BTV-8 was promoted, administration of fluvastatin did not fully protect mice.

We’ve previously shown that during the late stages of infection, NS4 is located in the cytoplasmic membrane of infected mammalian cells [12]. Our current findings demonstrate the significance of lipid pathways in bluetongue virus replication and suggest a more important role for membrane enveloped orbivirus particles than previously recognised.

## Figures and Tables

**Table 1:** Details of the six groups of IFNAR^(−/−)^ mice used in assessing potential antiviral effect of fluvastatin against bluetongue virus (BTV). PBS: Phosphate-buffered saline; PFU: Plaque forming unit.

Group	Fluvastatin Dose	Route of Administration	Days Administered	Challenge Virus
A	None (solvent diluted in PBS)	oral	Daily (days 0 to 12)	None
B	None (solvent diluted in PBS)	oral	Daily (days 0 to 12)	BTV-8 (10 PFU/mouse)
C	350 µg Fluvastatin/mouse (14.8 mg/kg/body weight: mouse-adapted dose)	oral	Daily (days 0 to 12)	None
D	28 µg Fluvastatin/mouse (1.2 mg/kg body weight: higher end human daily dose/kg body weight)	oral	Daily (days 0 to 7)	BTV-8 (10 PFU/mouse)
E	350 µg Fluvastatin/mouse	oral	Daily (days −1 to 12)	BTV-8 (10 PFU/mouse)
F	350 µg Fluvastatin/mouse	oral	Daily (days −1 to 12)	BTV-8 (10 PFU/mouse)

## Data Availability

All data are presented in the paper.

## Supplementary Materials

The following are available online at https://www.mdpi.com/article/10.3390/v13081437/s1, Figure S1: Identification and assembly of the mRNA sequence of *Culicoides sonorensis* HMG-CoA reductase, Table S1: Accession number of HMG-CoA reductases from various organisms used in construction of the tree shown in Figure 1, Table S2. Titres of YFV17D in cells treated with fluvastatin, inhibitors of geranyl-geranylation (geranyl-geranyl transferase inhibitor GGTI-2133, or farnesyl phosphate transferase inhibitor FTPIII), inhibitors of squalene synthase (zaragozic acid), or treated with fluvastatin then supplemented with components of the mevalonate pathway (mevalonic acid, geranyl-geranyl pyrophosphate, farnesyl pyrophosphate or cholesterol) to attempt restoring virus replication in fluvastatin-treated cells, Table S3. Titres of BTV-8 in BSR cells treated with fluvastatin, inhibitors of geranyl-geranylation (geranyl-geranyl transferase inhibitor GGTI-2133 or farnesyl phosphate transferase inhibitor FTPIII), inhibitors of squalene synthase (zaragozic acid A), or treated with fluvastatin then supplemented with components of the mevalonate pathway (mevalonic acid, geranylgeranyl pyrophosphate, farnesyl pyrophosphate or cholesterol) to attempt restoring virus replication in fluvastatin-treated cells, Table S4: Titres of BTV-8 in KC cells treated with fluvastatin, inhibitors of geranylgeranylation (geranylgeranyl transferase inhibitor GGTI-2133 and farnesyl phosphate transferase inhibitor FTPIII), inhibitors of squalene synthase (zaragozic acid A), or treated with fluvastatin then supplemented with mevalonic acid to attempt restoring virus replication in fluvastatin-treated cells, Table S5: Detection of BTV-8 RNA in blood samples of IFNAR(^−/−^) mice post-challenge/Fluvastatin treatment.
