# Supplementary material for: Inhibition of Orbivirus Replication by Fluvastatin and Identification of the Key Elements of the Mevalonate Pathway Involved
Source: Viruses. 2021 Jul 23;13(8):1437. doi: 10.3390/v13081437 (PMC8402872; doi:10.3390/v13081437)
Supplement: Supplementary file 1 [file viruses-13-01437-s001.zip › viruses-1300884-supplementary.pdf]

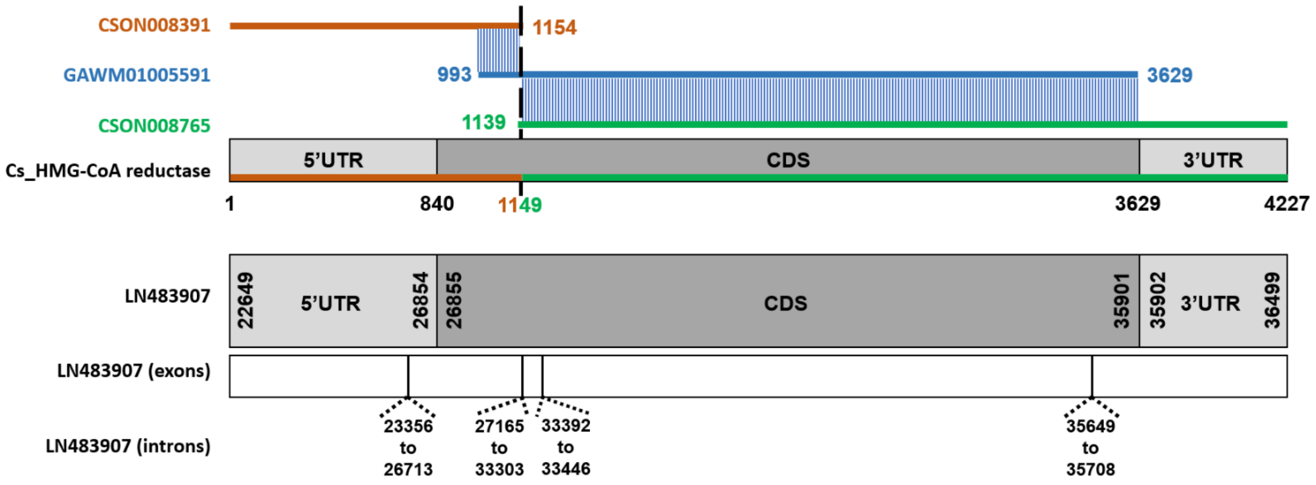

Figure S1. Identification and assembly of the mRNA sequence of *Culicoides sonorensis* HMG-CoA reductase. Transcripts CSON008391 and CSON008765 are only overlapping over a few nucleotides. The use of transcript GAWM0100559 (which overlaps with both CSON008391 and CSON008765) allows to correctly assemble the full-length sequence of *Culicoides sonorensis* HMG-CoA reductase. The validity of assembly is further corroborated by identification of this full-length sequence in a *Culicoides sonorensis* genome scaffold deposited in Genbank under accession number LN483907.

Table S1. Accession number of HMG-CoA reductases from various organisms used in construction of the tree shown in Figure 1.

Organism	Accession number	Protein length
Aedes aegypti	AAEL009311	930
Aedes albopictus	GCLM01025001	926
Anopheles gambiae	AGAP002288	924
Anopheles funestus	AFUN002171	924
Culex quinquefasciatus	CPIJ004077	907
Culex tarsalis	JAV24528	904
Culicoides sonorensis	CSON008391<br>CSON008765<br>GAWM01005591<br>LN483907	929
Drosophila melanogaster	AAA28608	916
Gallus gallus	XP_015132713	882
Homo sapiens	NP_000850	888
Ixodes persulcatus	GBXQ01000618	942
Ixodes ricinus	GIXL01023950	935
Ixodes scapularis	GHJT01004074	935
Lutzomyia longipalpis	MBC1168821	901
Lutzomia neivai	JAV11448	892
Mus musculus	NP_001347094	887
Ovis aries	XP_004010241	888
Phlebotomus kandelakii	NBJ57773	899
Salmo salar	XP_013998852	898

Table S2. Titres of YFV17D in cells treated with Fluvastatin, inhibitors of geranyl-geranylation (geranyl-geranyl transferase inhibitor GGTI-2133, or farnesyl phosphate transferase inhibitor FTPIII), inhibitors of squalene synthase (zaragozic acid), or treated with Fluvastatin then supplemented with components of the mevalonate pathway (mevalonic acid, geranyl-geranyl pyrophosphate, farnesyl pyrophosphate or cholesterol) to attempt restoring virus replication in Fluvastatin-treated cells.

Experiment	YFV17D	YFV17D Fluvastatin	YFV17D Fluvastatin mevalonic acid	YFV17D Fluvastatin geranylgeranyl pyrophosphate	YFV17D Fluvastatin farnesyl pyrophosphate	YFV17D Fluvastatin cholesterol	YFV17D GGTI-2133 FPTI III	YFV17D Zaragozic acid A
Mean 1 of triplicates	$5.1x10^7$	$7x10^5$	$1.1x10^7$	$7.4x10^6$	$1.6\ x10^6$	$9\ x10^6$	$2.9\ x10^7$	$7.1\ x10^6$
Mean 2 of triplicates	$3.9x10^7$	$5\ x10^5$	$7x10^6$	$8x10^6$	$1.8\ x10^6$	$2.7\ x10^6$	$2.3\ x10^7$	$9\ x10^6$
Mean 3 of triplicates	$3.6x10^7$	$5.5x10^5$	$9\ x10^6$	$7x10^6$	$2.3\ x10^6$	$4.8\ x10^6$	$3.1\ x10^7$	$7.3\ x10^6$
Mean 4 of triplicates	$4.3x10^7$	$4.9x10^5$	$1.2\ x10^7$	$1x10^7$	$2.8\ x10^6$	$3.3\ x10^6$	$1.6\ x10^7$	$5.9\ x10^6$
Mean overall	$4.2x10^7$	$5.6x10^5$	$9.75\ x10^6$	$8.1x10^6$	$2.12\ x10^6$	$4.95\ x10^6$	$2.47\ x10^7$	$7.32\ x10^6$
Log reduction (compared to YFV17D control)		1.85					0.2	0.8
Log recovery (compared to YFV17D treated with Fluvastatin)			1.24	1.17	0.6	0.96		

Table S3. Titres of BTV-8 in BSR cells treated with Fluvastatin, inhibitors of geranyl-geranylation (geranyl-geranyl transferase inhibitor GGTI-2133 or farnesyl phosphate transferase inhibitor FTPIII), inhibitors of squalene synthase (Zaragozic acid A), or treated with Fluvastatin then supplemented with components of the mevalonate pathway (mevalonic acid, geranylgeranyl pyrophosphate, farnesyl pyrophosphate or cholesterol) to attempt restoring virus replication in Fluvastatin-treated cells.

Experiment	BTV-8 control	BTV-8 Fluvastatin	BTV-8 Fluvastatin Mevalonate	BTV-8 Fluvastatin Geranylgeranyl pyrophosphate (GGPP)	BTV-8 Fluvastatin Farnesyl pyrophosphate (FPP)	BTV-8 Fluvastatin Cholesterol	BTV-8 Fluvastatin added then withdrawn	BTV-8 GGTI-2133	BTV-8 FPTI III	BTV-8 GGTI-2133 FPTI III	BTV-8 Zaragozic acid A
Mean 1 of triplicates	$2.8x10^8$	$1.1x\ x10^7$	$5.7\ x10^7$	$7.4\ x10^7$	$7.9\ x10^6$	$6.7\ x10^6$	$5.3x\ x10^7$	$1.8\ x10^7$	$8.6\ x10^6$	$6.3\ x10^6$	$9.9\ x10^7$
Mean 2 of triplicates	$3.1\ x10^8$	$3.6x10^6$	$7.8\ x10^7$	$1.6\ x10^7$	$4.1x10^6$	$5.9\ x10^6$	$7.1\ x10^7$	$7.6\ x10^6$	$3.2\ x10^6$	$3.2\ x10^6$	$6.4\ x10^7$
Mean 3 of triplicates	$3.9\ x10^8$	$9x10^6$	$1.5\ x10^8$	$9.9\ x10^7$	$9.1\ x10^6$	$1.3\ x10^7$	$5.5\ x10^7$	$5.9\ x10^6$	$6.5\ x10^6$	$9.2\ x10^6$	$4.1\ x10^7$
Mean 4 of triplicates	$2.9\ x10^8$	$4.9\ x10^6$	$6.9\ x10^7$	$4.3\ x10^7$	$5.2\ x10^6$	$4.6\ x10^6$	$4.3\ x10^7$	$9\ x10^6$	$1.4\ x10^7$	$7.6\ x10^6$	$5.3\ x10^7$
Mean overall	$3.2\ x10^8$	$7.1x10^6$	$8.9\ x10^7$	$5.8\ x10^7$	$6.6\ x10^6$	$7.4\ x10^6$	$5.6\ x10^7$	$1x10^7$	$8\ x10^6$	$6.6\ x10^6$	$6.4\ x10^7$
Log reduction (compared to BTV-8 control)		1.7	0.6	0.8	1.7	1.6	0.7	1.6	1.6	1.7	0.7
Log recovery (compared to BTV-8 treated with Fluvastatin)			1.1	0.9	0	0	0.9				

Table S4. Titres of BTV-8 in KC cells treated with Fluvastatin, inhibitors of geranylgeranylation (geranylgeranyl transferase inhibitor GGTI-2133 and farnesyl phosphate transferase inhibitor FTPIII), inhibitors of squalene synthase (Zaragozic acid A), or treated with Fluvastatin then supplemented with mevalonic acid to attempt restoring virus replication in Fluvastatin-treated cells. Titres were determined by plaque assay on BSR cells.

Experiment	BTV-8 control	BTV-8 Fluvastatin	BTV-8 Fluvastatin Mevalonate	BTV-8 GGTI-2133 FPTI III	BTV-8 Zaragozic acid A
Mean 1 of triplicates	$1.5x10^7$	$1.1\ x10^6$	$1\ x10^7$	$4.2\ x10^6$	$1.2\ x10^7$
Mean 2 of triplicates	$9\ x10^6$	$2.8\ x10^5$	$4.5\ x10^6$	$1.6\ x10^6$	$8x10^6$
Mean 3 of triplicates	$1.2\ x10^7$	$1\ x10^6$	$9.9\ x10^6$	$2.3\ x10^6$	$1\ x10^7$
Mean overall	$1.2\ x10^7$	$7.9\ x10^5$	$8.1\ x10^6$	$2.7\ x10^6$	$1\ x10^7$
Log reduction (compared to BTV-8 control)		1.2		0.65	0.1
Log recovery (compared to BTV-8 treated with Fluvastatin)			1		

Table S5. Detection of BTV-8 RNA in blood samples of IFNAR$^{(-/-)}$ mice post-challenge/Fluvastatin treatment. Ct values are shown for individual IFNAR$^{(-/-)}$ mice from the 6 groups used for assessing the potential antiviral effect of Fluvastatin on BTV-8. Comparison of groups C and E indicates that mice survival has been promoted, though only partial protection is achieved. A 3.3 Ct difference corresponds to a 10-fold (or 1 log) change in viral RNA copies.

Group	Mouse	Ct Day 4	Ct Day 6	Ct Day 8	Ct Day 12	Ct Day 15
	1	Neg	Neg	Neg	Neg	Neg
	2	Neg	Neg	Neg	Neg	Neg
A	3	Neg	Neg	Neg	Neg	Neg
	4	Neg	Neg	Neg	Neg	Neg
	5	Neg	Neg	Neg	Neg	Neg
	1	Neg	Neg	Neg	Neg	Neg
	2	Neg	Neg	Neg	Neg	Neg
B	3	Neg	Neg	Neg	Neg	Neg
	4	Neg	Neg	Neg	Neg	Neg
	5	Neg	Neg	Neg	Neg	Neg
	1	25.7	Died D5			
	2	28.1	Died D5			
C	3	24.6 [1]				
	4	26.5	31.8 [1]			
	5	27.4	Died D5			
	1	26.3	Died D5			
	2	27.6	33.4 [1]			
D	3	27.2	31.8 [1]			
	4	23.9	28.6			
	5	24.3	26.5	Died D7		
	1	29.8	34.7	Died D8		
	2	31.3	Died D5			
E	3	33.1	30.1	39.7		
	4	30.6	33.8	Died D8		
	5	34.6	36.9	Died D8		
	1	27 .4	Died D6			
	2	28.7	33.6	28.4 [2]		
F	3	25.5	35.1	Died D8		
	4	26.1	Died D6			
	5	23.4	Died D6			

[1] Euthanised the same day. [2] Euthanised the day after
